# Diagnostic value of high-resolution ultrasound combined with multi-slice computer tomography (MSCT) for pediatric intra-abdominal hernias: a retrospective study

**DOI:** 10.1186/s12893-024-02478-0

**Published:** 2024-06-17

**Authors:** Lichun Hua, Yaqing Huang, Hui Liu, Jun Chen, Ying Tang

**Affiliations:** 1 Department of Ultrasound, Children’s Hospital of Nanjing Medical University, No. 88, Jiangdong East Road, Jianye District, Nanjing, Jiangsu P.R. China; 2 Department of Radiology, Children’s Hospital of Nanjing Medical University, No. 88, Jiangdong East Road, Jianye District, Nanjing, Jiangsu P.R. China

**Keywords:** Ultrasound, Radiological imaging, Internal hernia, Child, Retrospective study

## Abstract

**Introduction:**

To explore the diagnostic value of high-resolution ultrasound combined with multi-slice computer tomography (MSCT) for pediatric intra-abdominal hernias (IAHs), and to analyze the potential causes for missed diagnosis and misdiagnosis of IAHs in children.

**Methods:**

A retrospective analysis was conducted on 45 children with surgically confirmed IAHs. The diagnostic rate of IAHs by preoperative high-resolution ultrasound combined with MSCT was compared with that of intraoperative examination, and the potential causes for missed diagnosis and misdiagnosis by the combination method were analyzed.

**Results:**

Forty-five cases of pediatric IAHs were categorized into primary (25/45, 55.5%) and acquired secondary hernias (20/45, 44.5%). Among children with primary hernias, mesenteric defects were identified as the predominant subtype (40%). Acquired secondary hernias typically resulted from abnormal openings in the abdominal wall or band adhesions due to trauma, surgery, or inflammation. In particular, adhesive band hernias were the major type in children with acquired secondary hernias (40%). The diagnostic rate of high-resolution ultrasound was 77.8%, with “cross sign” as a characteristic ultrasonic feature. Among 10 cases of missed diagnosis or misdiagnosis, 5 were finally diagnosed as IAHs by multi-slice computer tomography (MSCT). Overall, the diagnostic rate of pediatric IAHs by preoperative ultrasound combined with radiological imaging reached 88.9%.

**Discussion:**

IAHs in children, particularly mesenteric defects, are prone to strangulated intestinal obstruction and necrosis. High-resolution ultrasound combined with MSCT greatly enhances the diagnostic accuracy of pediatric IAHs.

**Supplementary Information:**

The online version contains supplementary material available at 10.1186/s12893-024-02478-0.

## Introduction

An intra-abdominal hernia (IAH) is defined as a protrusion of abdominal viscera, most commonly small bowel loops, through a peritoneal or mesenteric aperture into an abdominal or pelvic compartment [1]. IAHs can be divided into primary and secondary. Although presenting a low incidence, IAHs account for 1% of all causes of intestinal obstruction and a high proportion of strangulated hernias. IAHs can develop rapidly and become serious enough to result in strangulated intestinal obstruction, intestinal wall necrosis, perforation, abdominal infection and even death [2]. Early diagnosis and treatment are essential to improve the prognosis of IAHs, although the preoperative diagnosis by imaging techniques is challenging [3]. In the present study, we calculated the diagnostic rate of preoperative high-resolution ultrasound combined with multi-slice computer tomography (MSCT) in pediatric IAHs, and analyzed the potential causes for its missed diagnosis and misdiagnosis.

## Materials and methods

### Subjects

This was a retrospective analysis involving 45 cases of pediatric IAHs confirmed through surgery and postoperative pathology in Nanjing Children’s Hospital from April 2017 to April 2022. Demographic characteristics, major clinical manifestations, onset time, preoperative imaging findings, intraoperative observations, and postoperative recovery were recorded. All patients underwent preoperative ultrasound and upright abdominal radiography, and 35 cases were examined by MSCT.

### Ultrasonography

Used in this study were the GE Logiq E8 ultrasonic machine with a high-frequency 9 L transducer (8–12 MHz) and a low-frequency C1-6 transducer (1–6 MHz), Philips IU22 ultrasonic instrument with a high-frequency L12-5 transducer (5–11 MHz) and a low- frequency C8-5 transducer (5–8 MHz), and Esaote ultrasonic instrument with LA523 a transducer (4–13 MHz).

In a supine position, patients were instructed to remain calm; otherwise, oral or rectal administration of 5% hydrated chloral hydrate was applied to those with a poor cooperation. A longitudinal midline scan below the xiphoid process was performed to visualize the lower esophagus and lower esophageal sphincter, and a transverse scan below the xiphoid process was then conducted to assess the gastric cavity, pylorus, duodenum, and initial segment of the jejunum. The remaining small intestine was thoroughly scanned from left to right and from top to bottom. In the right lower abdomen, colorectal segments were carefully scanned along the colonic frames, highlighting the examination at the junction between dilated and collapsed intestinal segments. Notably, the degree of intestinal distension, presence of gas or fluid accumulation, thickness and layering of the intestinal wall, and any signs of ischemic changes were recorded. Intestinal peristalsis with any abnormal slowing or absence of movement was dynamically observed. Furthermore, adhesions, masses, or bands between the intestinal wall and adjacent structures (e.g., the abdominal wall or mesentery) was observed [4, 5].

A routine scanning of other organs, such as the liver, gallbladder, uterus, bilateral ovaries in females, and the inguinal region and scrotum in males was performed to exclude any potential acute abdominal conditions (e.g., ovarian or testicular torsion).

Ultrasonography was performed by an attending ultrasonographer equipped with over 10 years of clinical experiences, and positive findings were reviewed by a senior physician possessing a high-level professional title. They finally determined the ultrasound diagnosis through discussion.

### Statistical analysis

Data analysis was conducted using SPSS 22.0 software. Fisher’s exact test and Chi-square test were applied to compare categorical data, while the *t*-test to compare continuous data. The receiver operating characteristic (ROC) curve and the area under the curve (AUC) were used to assess the diagnostic potential. *P* < 0.05 was considered as statistically significant.

## Results

### Baseline characteristics of participants

**Fig. 1:**
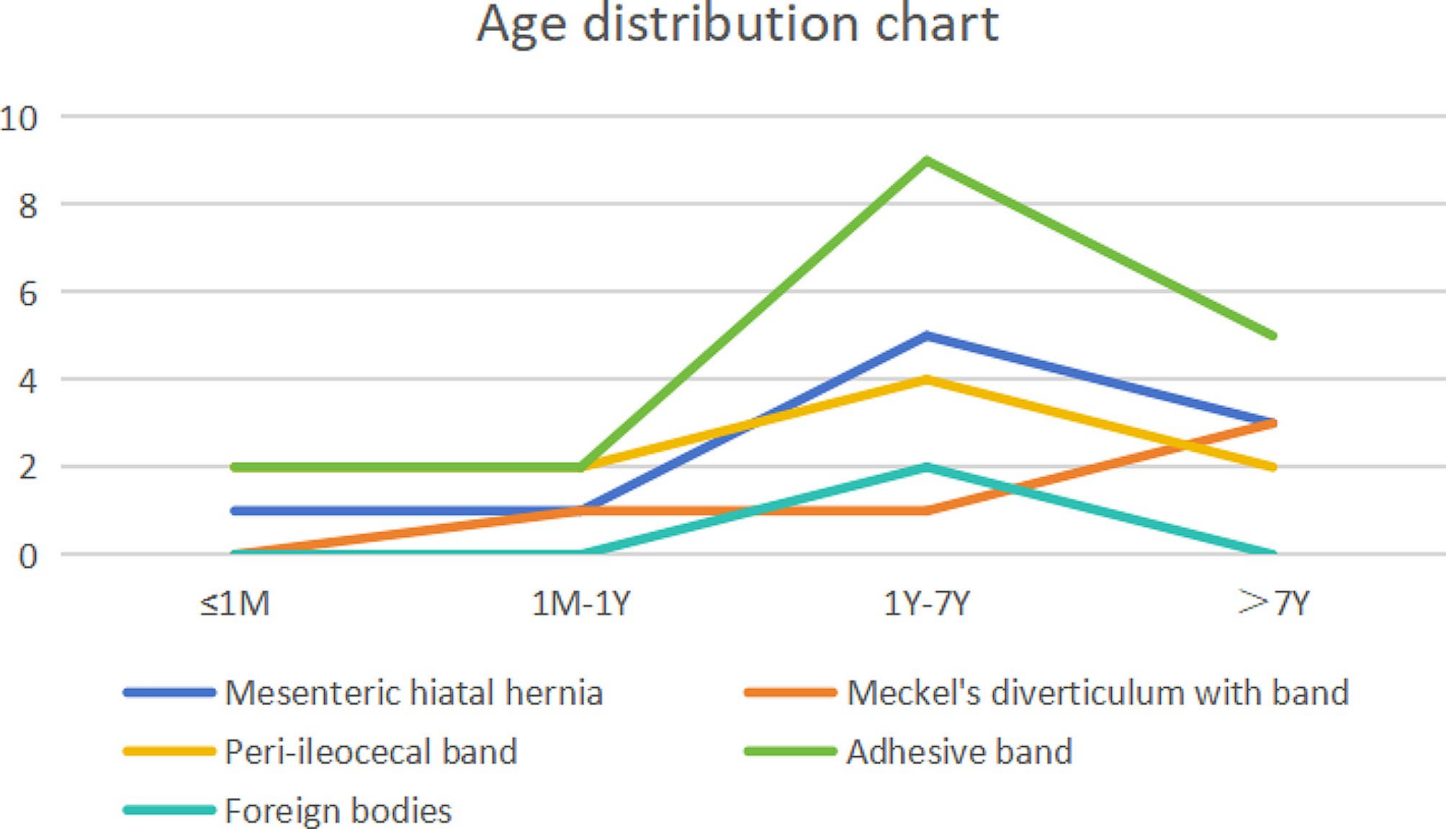
Age distribution of children with different types of IAHs.

A total of 45 children with a mean age of 5.3 ± 4.6 (from 3 h to 15 years) were enrolled into the present study, including 26 males and 19 females. They presented digestive symptoms, such as abdominal pain, diarrhea, bloating, vomiting, and bloody stools. Congenital IAHs were reported in 25 children, including 10 cases of mesenteric defects, 5 cases caused by mesodiverticular band of Meckel’s diverticulum, and 10 cases caused by ileocecal mesenteric defects or adhesive bands. Acquired secondary hernias were detected in 20 cases, including 18 cases of adhesive band hernias (history of abdominal surgery reported in 11 cases) and 2 cases of IAHs caused by ingestion of magnetic foreign bodies (Table 1). Age distribution with IAH type is shown in Fig. 1. There were no significant differences in age, gender and clinical manifestations between children with primary and secondary acquired IAHs (*P* > 0.05).

**Table 1:** Baseline clinical features of the different subtypes population

Type	Cases(%)	Mean values of age(years)	Male/Female	Clinical symptoms				Death count
				Abdominal pain	vomiting	fever	hematuria	
Mesenteric hiatal hernia	10(22.22%)	4.9 ± 3.2(1d-10y)	4/6	8	9	7	3	1
Meckel’s diverticulum with band	5(11.11%)	7.5 ± 4.6 (1y-13y)	4/1	5	5	1	1	0
Peri-ileocecal band	10(22.22%)	3.7 ± 4.9 (5 h-14.6y)	6/4	9	5	2	0	0
Adhesive band	18(40%)	6.4 ± 5.2 (1d-16y)	10/8	15	15	7	1	0
Foreign bodies	2(4.45%)	2.3 ± 0.7 (1.8y,2.8y)	2/0	2	1	0	0	0
*P* value		0.38	0.53	0.95	0.16	0.11	0.17	

### Diagnosis of IAHs by ultrasound

Preoperative ultrasonography recognized 35 cases of pediatric IAHs, with a diagnostic rate of 77.8%.

**Fig. 2:**
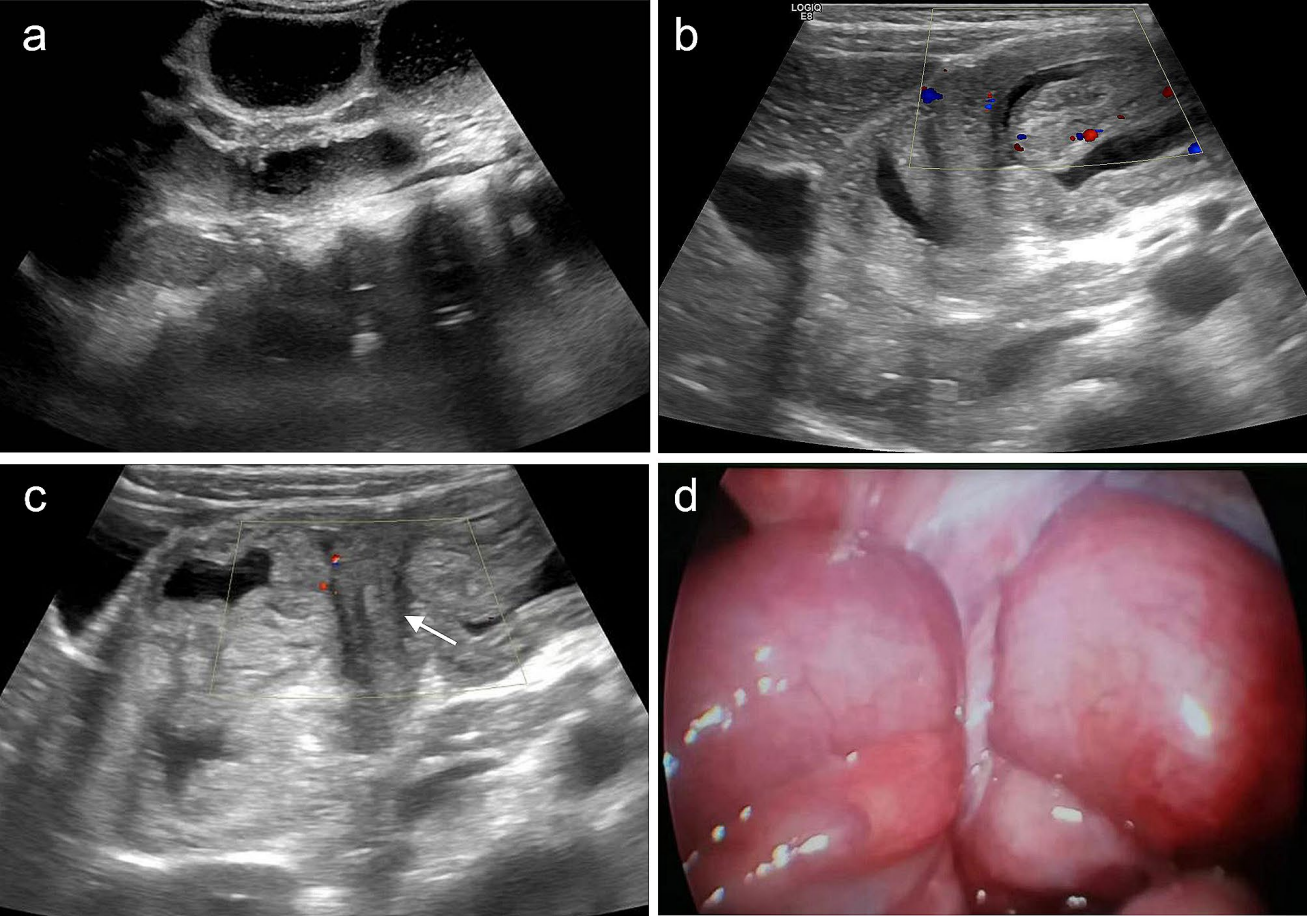
Representative imaging scans of a 5-year-old female patient with an IAH. **A**: Fluid accumulation in the small bowel loops visualized on the ultrasound scan, considering the intestinal obstruction. **B**: Ultrasound scan showing significant edema and thickening of the intestinal wall, and CDFI showing a reduced blood supply. Postoperative diagnosis of an IAH caused by adhesive band and secondary intestinal obstruction was made. **C**: Postoperative appendicitis, adhesive bands and the herniated bowel were visualized. The wide low echo (white arrow), short adhesive bands and dilation of the intestinal tubes at both ends constitute a typical “cross sign”. **D**: Postoperative appendicitis, adhesive banding with the greater omentum (white arrow) and dilation of the herniated intestine at both ends were shown

**Fig. 3:**
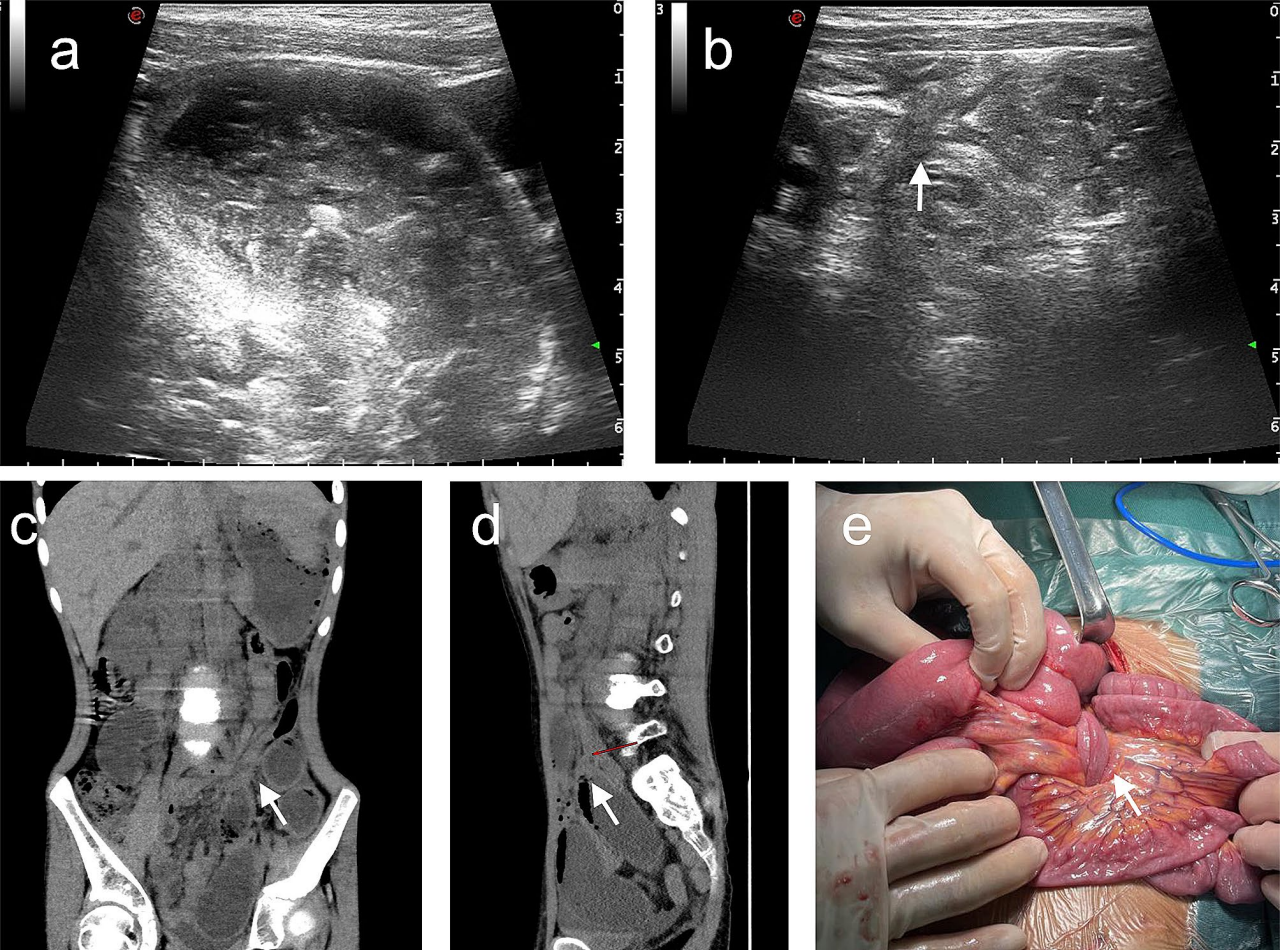
Representative imaging scans of a 7-year-old male patient presenting abdominal pain. **A**, **B**: Ultrasound scans visualizing an IAH complicated with intestinal dilation and obstruction, thickening of the intestinal wall, liquid exudation in the surrounding intestinal space, and a “cross sign” ( white arrow) of the adhesive bands in the dilated intestinal tubes. **C**, **D**: Coronal and sagittal CT scans of the abdomen showed dilatation of intestinal cavity in the left middle and lower abdomen, intestinal effusion, thickening of intestinal wall and visible gas accumulation. The obstruction point (white arrow) was visualized, where the intestinal tube was entangled with the hyperechoic mesentery. **E**: Intraoperative manifestations of an IAH, including the banding between the beginning of jejunum and the ileum, twisted proximal intestine, extensive adhesion between the small intestine, and a C ring at the adhesive bands (white arrow)

A panel of ultrasonic manifestations were observed in children with IAHs. First, ultrasound evidence referred fluid accumulation in the dilated intestine, thickening of the intestinal wall, and decreased blood supply to the intestinal wall (Figs. 2A and B and 3A). Second, a cord-like hypoecho was observed at the junction of dilated and withered intestine. Color Doppler flow imaging (CDFI) showed a visible blood flow signal in the cord-like hypoecho, and no blood flow signal was observed in the adhesion zone (Fig. 2B, C). The hernial ring intersected with the herniated inguinal canal formed a typical “cross sign” (Figs. 2C and D and 3B) [6, 7]. Third, a beak sign was seen in IAH patients with closed-loop obstruction caused by tapering bowel loops. The double beak sign described two or more collapsed loops just at the site of intestinal herniation. Fourth, the thickened mesentery thickened and dilated mesenteric vein were observed on ultrasound scans. Turbid ascites could also be found.

**Fig. 4:**
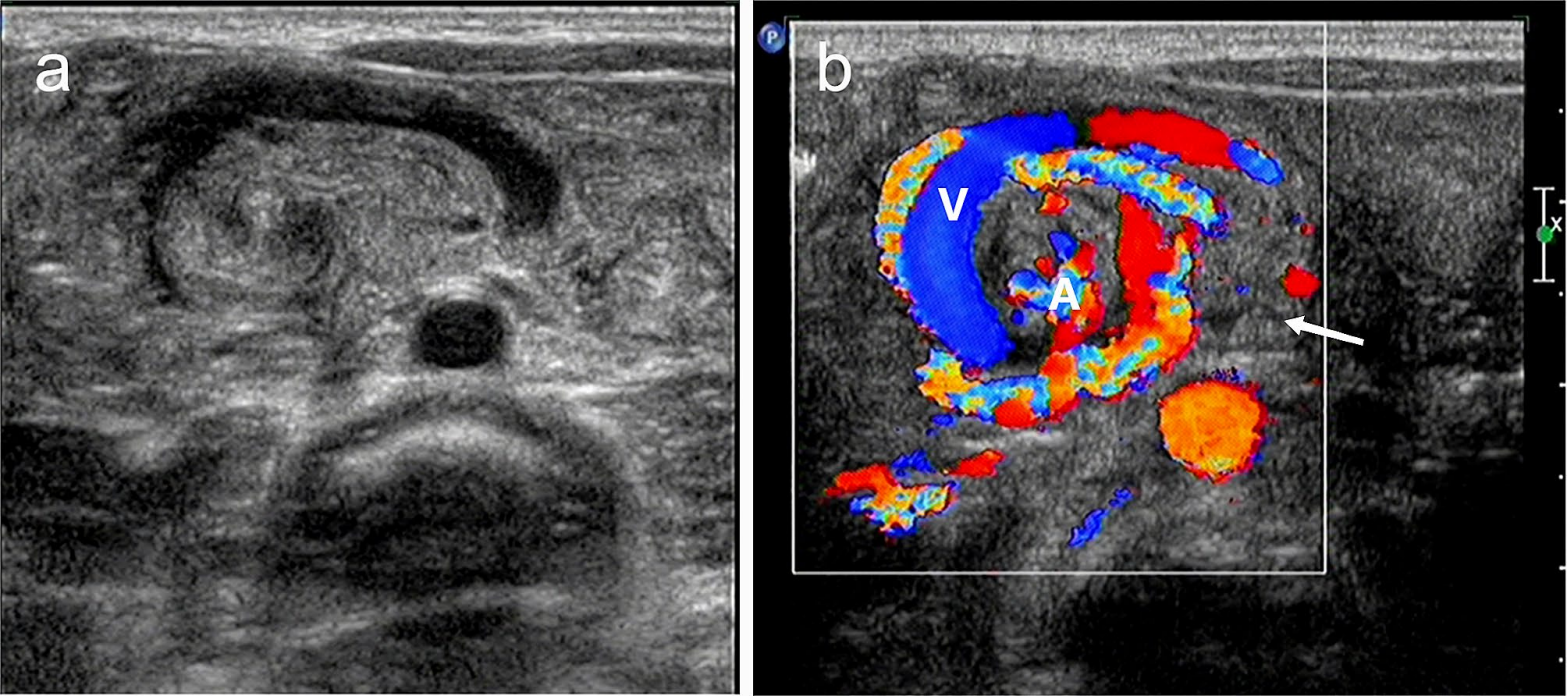
Representative imaging scans of a 2-year-old male patient presenting vomiting and abdominal pain. **A**: Congenital malrotation of the intestine with midgut volvulus was observed. The cross-sectional color doppler ultrasound image visualizing a clockwise twisting of the superior mesenteric vein (SMV) around the superior mesenteric artery (SMA) at the center of the intestinal twist, and a target ring of an isoechoic mass (white arrow). **B**: CDFI showed a swirling sign centered on the SMA with red and blue blood flow signals. Congenital malrotation of the intestine with midgut volvulus was postoperative confirmed. The greater omentum and the distal mesentery were tightly adherent to form a bundle of intraperitoneal hernia.

**Fig. 5:**
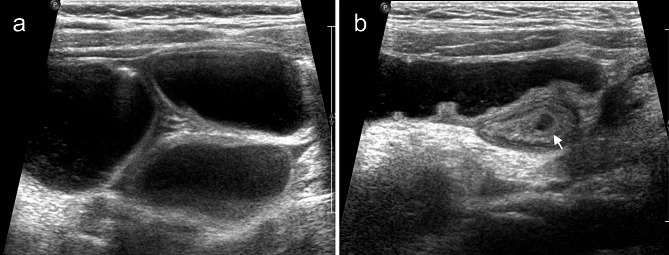
Representative imaging scans of a 7-year-old male patient presenting vomiting and abdominal pain. The ultrasound scan visualized Mekel’s diverticulum (white arrow), dilation of multiple segments of the small intestine and fluid accumulation in the abdominal cavity. Postoperative diagnosis of an IAH caused by Meckel’s diverticulum banding and secondary intestinal obstruction was made.

Other complications were detected by ultrasound as follows. Congenital intestinal malrotation with midgut volvulus on diagnostic imaging was characterized by the superior mesenteric vein (SMV) rotated around the superior mesenteric artery (SMA), with a target ring of an isoechoic mass (Fig. 4A, B). CDFI showed the swirling sign centered on the SMA, with red and blue colors of blood flow signals. Meckel’s diverticulum was visualized on ultrasound scans as an abnormal connection to the intestine, and the perforation caused the formation of an abscess around (Fig. 5A, B). Imaging signs of obstructions included small bowel stricture, atresia, dilation in the proximal intestine, and effusion in the distal end, meconium peritonitis and pseudocysts. Abdominal calcification and effusion could be seen in patients with the complication of intestinal perforation. In 2 cases of IAHs caused by ingestion of magnetic bead foreign bodies, ultrasound examination revealed a ring-like structure of adhesive foreign bodies within multiple intestinal segments, resulting in intestinal compression and adhesions. Additionally, fluid dilatation and a rigid appearance were visualized in the upper part of the intestine.

A total of 28 children were diagnosed with strangulating bowel obstruction by preoperative high-resolution ultrasound, manifesting ischemic changes in the thickening intestinal wall, high tension, echo reduction and blurred hierarchical structure. CDFI showed a significantly reduced blood flow in the intestinal wall, and thickening and adhesion of the surrounding mesentery (Fig. 2B).

### Misdiagnosis and missed diagnosis of IAHs by ultrasound

There were 6 (13.3%) cases of misdiagnosis and 4 (8.9%) cases of missed diagnosis. Two cases of adhesive internal hernias without a history of abdominal surgery and high intra-intestinal tension were misdiagnosed as incomplete intestinal obstructions, and the obstructed point and adhesive bands were not identified during preoperative diagnosis. Two cases of intraperitoneal hernias with peri-ileocecal bands were misdiagnosed as abdominal distension due to abdominal distension and crying. A case of IAH caused by Meckel’s diverticulum was misdiagnosed as an appendiceal abscess due to diffuse inflammation caused by the perforated Meckel’s diverticulum. One case of IAH caused by ingestion of magnetic foreign bodies was misdiagnosed as bowel obstruction due to mechanical obstruction of the disordered intestine.

Intestinal malformations were the major cause for missed diagnosis of IAHs by ultrasound. In detail, one case of mesenteric hernia through a foramen with a 720-degree twisting of the ileum was initially diagnosed as intestinal volvulus. One case of mesenteric hernia through a foramen with congenital intestinal malrotation and midgut volvulus was initially diagnosed as malrotation. One case of ileal atresia with secondary adhesive internal hernia was initially diagnosed as suspected intestinal atresia, due to diffuse peritonitis and echogenicity within the abdominal cavity. One case of congenital stenosis of the small intestine with inflammation and secondary adhesive internal hernia was initially diagnosed as inflammatory stenosis of the small intestine.

## Other radiological imaging findings

All patients were examined by abdominal X-ray, involving 37 cases of intestinal obstruction, 4 cases of bowel distension, 2 cases of foreign body obstruction, and 2 cases of necrotizing enterocolitis (NEC). However, a direct indication of IAHs was not available on X-ray scans. Among the 35 cases examined by MSCT, 28 cases were diagnosed with intestinal obstructions. Internal hernias were indicated in 13 cases presenting strangulated intestinal obstructions on MSCT scans. Overall, intestinal obstruction was accurately diagnosed by MSCT in 80% of cases, but internal hernia only in 37.1%.

### Diagnosis of IAHs by preoperative ultrasound combined with radiological imaging

**Fig. 6:**
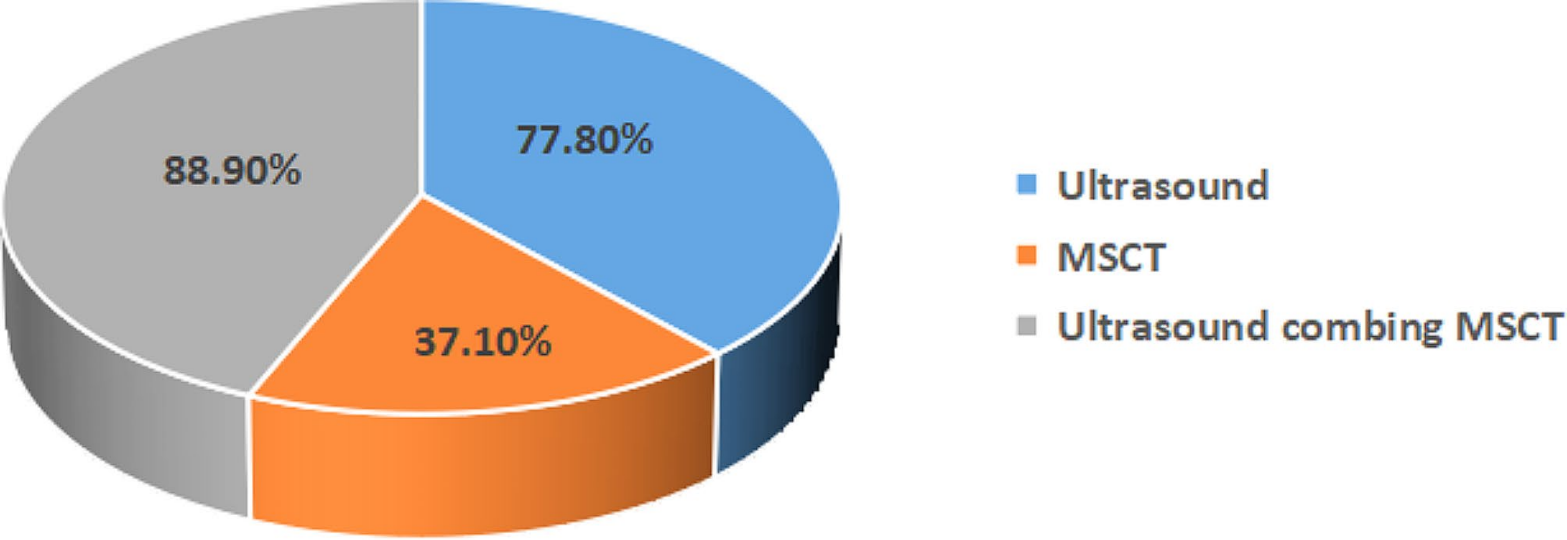
Diagnostic accuracy in pediatric IAHs by different imaging techniques

Among the 10 cases of misdiagnosis and missed diagnosis by ultrasound, 5 patients were finally diagnosed as IAHs by MSCT. As a result, the diagnostic rate of pediatric IAHs by preoperative ultrasound combined with MSCT was inflated to 88.9%. Typical MSCT signs of IAHs included images of intestinal obstruction, ischemia and necrosis. First, signs of intestinal obstruction on MSCT scans were composed of dilated lumen of the proximal intestine due to effusion, collapse and occlusion of distal intestine, and the presence of a beak sign (abnormal movement of the intestine, mesentery and corresponding blood vessels, and sudden stenosis of the dilated intestine with beak-like changes). Second, IAH-induced intestinal ischemia were radiologically characterized by intestinal wall edema and thickening, abnormal enhancement of the intestinal wall, ascites, cloud or swirl signs on MSCT scans. Third, IAH-caused intestinal necrosis visualized on MSCT scans presented an increased density in mesenteric vessels and intestinal wall, no enhancement of the intestinal wall, and gas in the intestinal wall or mesentery (Fig. 3C, D). We detected a significant difference in the diagnostic rate of pediatric IAHs by preoperative high-resolution ultrasound, MSCT and their combination (χ^2^ = 23.1, *P* < 0.01, Fig. 6). The combined method offered a highest accuracy in diagnosing IAHs in children.

**Fig. 7:**
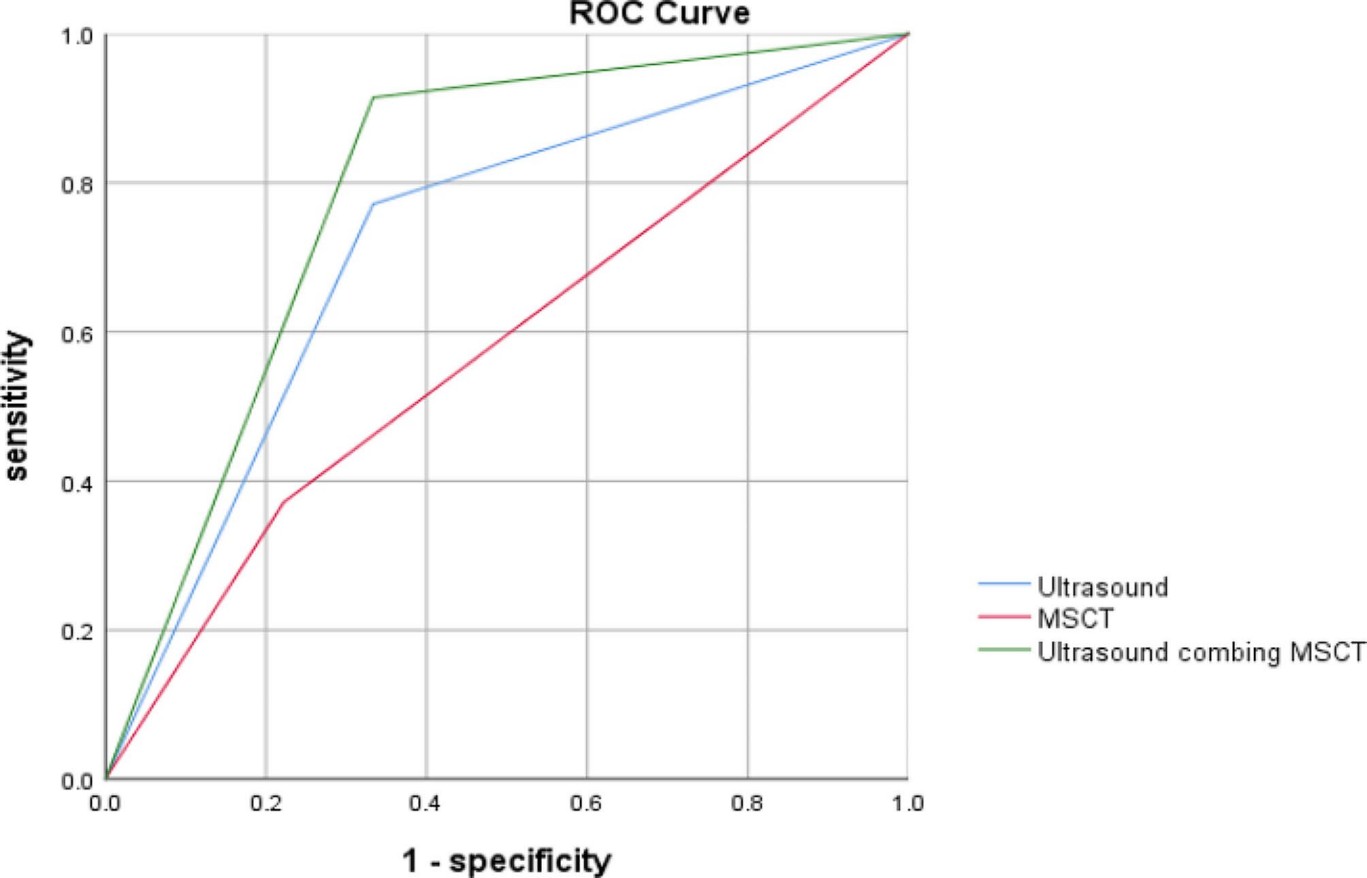
ROC curves of imaging techniques in discriminating pediatric IAHs.

To validate our findings, we conducted a retrospective analysis involving 60 children with intestinal obstruction possibly caused by IAHs during the same period. According to the conclusive surgical outcomes, preoperative ultrasound combined with MSCT yielded the largest AUC (AUC = 0.79) in discriminating IAHs in children, compared to a single examination (AUC = 0.719 for ultrasound and AUC = 0.575 for MSCT, Fig. 7).

### Operation results

**Fig. 8:**
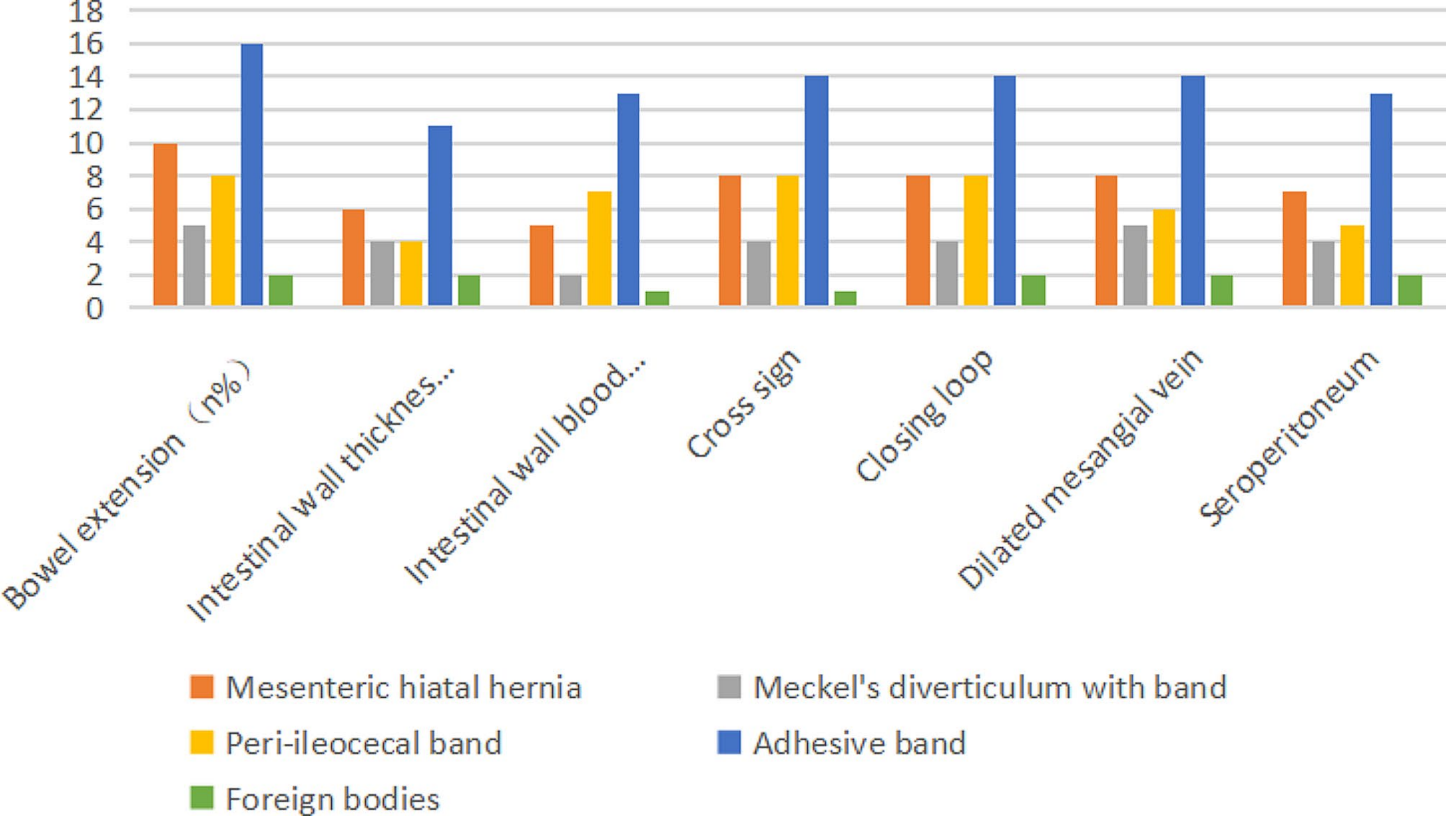
Histogram of ultrasound features of different subtypes of IAHs.

All 45 children with IAHs were surgically treated. No significant differences in ultrasonic findings were detected in children with different subtypes of IAHs (*P* > 0.05, Fig. 8).

Among the 10 IAH patients caused by mesenteric defects, 5 with intestinal ischemia and necrosis due to a long-term protrusion of the intestine were surgically treated with intestinal resection and anastomosis, and the other 5 with intestinal reduction. Fifteen IAH patients caused by mesodiverticular band of Meckel’s diverticulum, ileocecal mesenteric defects or adhesive bands were surgically removed, although the perforation of the inflamed diverticula formed an abscess.

Among the 18 patients with acquired secondary hernias caused by adhesive bands, 11 with a history of surgery suffered from postoperative adhesions. The remaining 7 cases without a history of surgery included 2 neonates with intestinal atresia and stricture, 2 cases of meconium peritonitis after intestinal perforation and adhesive bands formed by intestinal perforation and necrosis, 2 cases of the formation of adhesive bands by Kawasaki disease, and 1 case of congenital intestinal malrotation with midgut volvulus and the greater omentum densely adherent to the distal mesentery. For surgically treated 18 children with acquired secondary hernias, 17 were managed by the lysis of intestinal adhesions and reduction, and 1 by the Ladd’s procedure. Ingested magnet beads were surgically removed in 2 cases.

Forty-four children were discharged after surgery, and one suffered from postoperative strangulating intestinal obstruction, long-term herniation, necrosis of multiple intestinal segments and multiple organ failure. Divided by the accurate or inaccurate ultrasound diagnosis, postoperative complications and outcomes were compared between groups, including the time from presentation to surgery, length of stay, incidence of bowel resection, length of bowel resection, and incidence of short bowel syndrome (SBS). Significant differences were observed in the length of bowel resection and incidence of SBS between IAH children accurately and inaccurately diagnosed by preoperative ultrasound (*P* < 0.05 Table 2).

**Table 2:** Comparative analysis of clinical hospitalization indicators among different ultrasonic examination results

Ultrasonic examination results	Time from presentation to surgery(days)	Hospitalization duration(days)	Length of bowel resected (cm)	Bowl resection rate (%)	SBS rate (%)
Correct diagnosis	1.00 ± 0.12	15.0 ± 1.70	49.0 ± 13.47	15/35	5/35
Incorrect diagnosis	1.80 ± 0.44	15.0 ± 2.60	19.71 ± 5.97	7/10	5/10
Statistic value	*t = 1.75*	*t = 0.29*	*t = 2.21*	*χ*^*2*^ *= 2.29*	*χ* ^*2*^ *=5.74*
*P* value	0.11	0.98	0.03*	0.13	0.017*

*indicates a *P* value of < 0.05, with a statistically significant difference

## Discussion

IAHs can be divided into primary and secondary. Primary IAHs are believed as the consequences of the protrusion of an organ into congenital abdominal compartment due to intestinal rotation and peritoneal attachment during the embryonic period. Primary IAHs are represented by hiatal hernias (mesenteric defects), omental hernias, peritoneal hernias and other congenital IAHs caused by adhesive bands. In this study, mesenteric defects were the major causes for congenital IAHs (40%), which is consistent with previously reported statistics [7]. Hiatal hernias (mesenteric defects) usually occur in the distal ileal mesentery mesenteric ileum, featured by more intestinal tubes bulging into the herniation, low possibility of congenital intestinal stenosis, high risk of strangulating intestinal obstruction, and early onset and serious conditions in children. One child with mesenteric defects died of IAH.

Meckel’s diverticulum, mesangial and ileocecal banding are all congenital. Meckel’s diverticulum with mesodiverticular band is formed by a part of the intestine bulging into the fibrous band between the tip of the diverticula and the umbilical cord that does not completely close off during fetal development, leading to high risks of perforation, massive inflammation, intestinal adhesions and formation of abscesses [8]. Bearing similar imaging features with those of adhesive bowel obstruction secondary to appendicitis, IAHs caused by mesangial or ileocecal banding are easily misdiagnosed as appendiceal abscesses at the same site in the right lower abdomen. Acquired secondary IAHs are mainly caused by trauma, surgery, inflammation, postoperative adhesions of the gastrointestinal tract and intraperitoneal adhesive bands [9, 10]. In this study, the incidence of pediatric IAHs caused by adhesive banding was up to 40%. The relatively high incidence may be attributed to a previous history of abdominal surgery.

A variety of imaging techniques are available to diagnose IAHs, although atypical clinical and imaging manifestations obstruct the way to make an accurate diagnosis [11, 12]. Upper digestive tract angiography and MSCT have a high accuracy in diagnosing paraduodenal hernias [13], but they provide an inferior performance in the diagnosis of other subtypes of hernias [14]. Boasting of non-ionizing radiation, clear visualization, simple procedures and low price, high-resolution ultrasound is preferred to preoperative screening of IAHs [15]. In early-stage IAHs when bowel dilation is not obvious, ultrasound imaging presents limited ability to recognize hernias. With the development of mechanical intestinal obstruction, ultrasound features of reduced blood supply to the intestinal wall, intestinal wall edema and thickening and inflammatory responses at various degrees are all indicative of an IAH. In serious cases like strangulating intestinal obstruction, ultrasound visualization of no obvious blood supply, necrosis of the intestinal wall and gas accumulation provide useful diagnostic information. In tracing along the dilated intestinal duct to the collapsed intestinal duct, a typical “cross sign” of a cord-like hypoecho can be seen at the obstruction point of the IAH, with the hernial ring intersected with the intestinal duct [16].

In this study, the accuracy of pediatric IAHs by preoperative high-resolution ultrasound was much lower than the previously reported (77.8% vs. 91.7%), which may be related to different sample sizes and clinical experiences of sonographers. Among the 10 cases of missed diagnosis and misdiagnosis, 5 were further confirmed by MSCT. The combination of MSCT with preoperative ultrasound increased the diagnostic rate to 88.9%. MSCT is especially sensitive in detecting abdominal fluid and intestinal wall abnormalities, enabling an early diagnosis and timely intervention to improve the prognosis. An accurate preoperative imaging diagnosis of IAHs is the cornerstone to make an optimal surgical decision, which significantly influences the length of bowel resection and the incidence of SBS.

Based on our clinical experiences and literature review, we recommended the following measures to prevent the missed diagnosis and misdiagnosis of pediatric IAHs. First, imaging features of early-stage IAHs are atypical and similar to those of incomplete intestinal obstruction. We therefore recommended other auxiliary examinations to assist the diagnosis of IAHs. Second, ultrasound scanning in multiple sections of each intestinal segment is essential to prevent missed diagnosis and misdiagnosis of IAHs [17, 18]. Third, congenital intestinal malformation is a vital factor shadowing an accurate imaging diagnosis of IAHs [19–21]. Fourth, IAHs caused by ingestion of foreign bodies are rare but highly alarming. The possibility of an IAH should be concerned during the procedure for foreign body removal. Last, consensus standards for imaging modalities for pediatric IAHs are urgently needed to increase the diagnostic rate. Any uncertainties are recommended to be solved by discussion with a multidisciplinary team [22].

## Conclusions

Preoperative high-resolution ultrasound combined with MSCT greatly increases the diagnostic rate of pediatric IAHs. An accurate preoperative diagnosis of IAHs by imaging examinations favors the surgical outcomes, especially in lowering the length of bowel resection and the incidence of SBS.

### Electronic supplementary material

Below is the link to the electronic supplementary material.

Supplementary Material 1

## Data Availability

The data used to support the findings of this study are available from the cor-responding author on reasonable request.
